# Supplementary material for: Adherence to the Mediterranean Diet and Health-Related Quality of Life during the COVID-19 Lockdown: A Cross-Sectional Study including Preschoolers, Children, and Adolescents from Brazil and Spain
Source: Nutrients. 2023 Jan 29;15(3):677. doi: 10.3390/nu15030677 (PMC9920644; doi:10.3390/nu15030677)
Supplement: Supplementary file 1 [file nutrients-15-00677-s001.zip › Table S1.pdf]

Table S1. Mediterranean Diet Quality Index in Children and Adolescents.

**Scoring**
Takes a fruit or fruit juice every day (+1)
Has a second fruit every day (+1)
Has fresh or cooked vegetables regularly once a day (+1)
Has fresh or cooked vegetables more than once a day (+1)
Consumes fish regularly (at least 2–3 times per week) (+1)
Goes more than once a week to a fast-food (hamburger) restaurant (−1)
Likes pulses and eats them more than once a week (+1)
Consumes pasta or rice almost every day (5 or more times per week)
Has cereals or grains (bread, etc.) for breakfast (+1)
Consumes nuts regularly (at least 2–3 times per week) (+1)
Uses olive oil at home (+1)
Skips breakfast (−1)
Has a dairy product for breakfast (yoghurt, milk, etc.) (+1)
Has commercially baked goods or pastries for breakfast (−1)
Takes two yoghurts and/or some cheese (40 g) daily (+1)
Takes sweets and candy several times every day (−1)
