# Supplementary material for: Adherence to the Mediterranean Diet and Health-Related Quality of Life during the COVID-19 Lockdown: A Cross-Sectional Study including Preschoolers, Children, and Adolescents from Brazil and Spain
Source: Nutrients. 2023 Jan 29;15(3):677. doi: 10.3390/nu15030677 (PMC9920644; doi:10.3390/nu15030677)
Supplement: Supplementary file 1 [file nutrients-15-00677-s001.zip › Table S2.pdf]

Table S2. Association between Mediterranean Diet adherence and problems in different dimensions of health-related quality of life by sex and age group.

HRQoL Domain [a]	Low/Moderate MD	High MD	*p*
**Sex**			
**Boys**			
Mobility	2 (5.6)	14 (2.6)	0.264
Looking after myself	3 (8.3)	57 (10.6)	0.673
Doing usual activities	7 (19.4)	53 (9.8)	0.067
Having pain or discomfort	10 (27.8)	84 (15.6)	0.055
Feeling worried, sad, or unhappy	13 (36.1)	214 (39.7)	0.669
**Girls**			
Mobility	-	12 (2.5)	1.000
Looking after myself	5 (12.8)	47 (9.7)	0.532
Doing usual activities	10 (25.6)	43 (8.9)	<0.001
Having pain or discomfort	13 (33.3)	74 (15.3)	0.004
Feeling worried, sad, or unhappy	19 (48.7)	191 (39.6)	0.266
**Age group**			
**Preschoolers**			
Mobility	-	6 (4.2)	1.000
Looking after myself	1 (50.0)	36 (25.0)	0.420
Doing usual activities	1 (50.0)	23 (16.0)	0.197
Having pain or discomfort	-	17 (11.8)	0.605
Feeling worried, sad, or unhappy	1 (50.0)	59 (41.3)	1.000
**Children**			
Mobility	1 (4.0)	6 (1.4)	0.335
Looking after myself	3 (12.0)	25 (6.0)	0.228
Doing usual activities	8 (32.0)	39 (9.3)	<0.001
Having pain or discomfort	8 (32.0)	48 (11.5)	0.003
Feeling worried, sad, or unhappy	12 (48.0)	154 (36.8)	0.259
**Adolescents**			
Mobility	1 (2.1)	14 (3.0)	1.000
Looking after myself	4 (8.3)	43 (9.3)	0.821
Doing usual activities	8 (16.7)	34 (7.4)	0.026
Having pain or discomfort	15 (31.3)	93 (20.2)	0.074
Feeling worried, sad, or unhappy	19 (39.6)	192 (41.8)	0.764

[a] Data expressed as proportion of participants reporting any problem in the different health-related quality of life domains according to adherence to the Mediterranean diet. HRQoL, health-related quality of life; MD, Mediterranean diet. P values obtained by Pearson's chi-square test or Fisher's exact test.
