# Supplementary figures and images for: Adherence to the Mediterranean Diet and Health-Related Quality of Life during the COVID-19 Lockdown: A Cross-Sectional Study including Preschoolers, Children, and Adolescents from Brazil and Spain
Source: Nutrients. 2023 Jan 29;15(3):677. doi: 10.3390/nu15030677 (PMC9920644; doi:10.3390/nu15030677)

**A**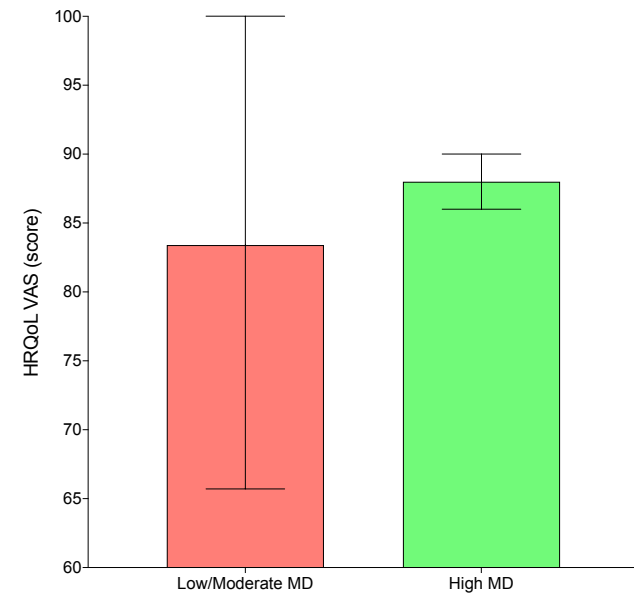**B**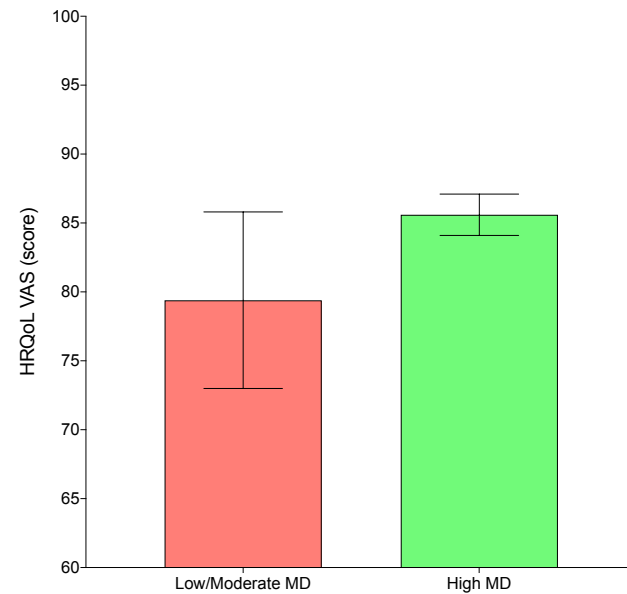**C**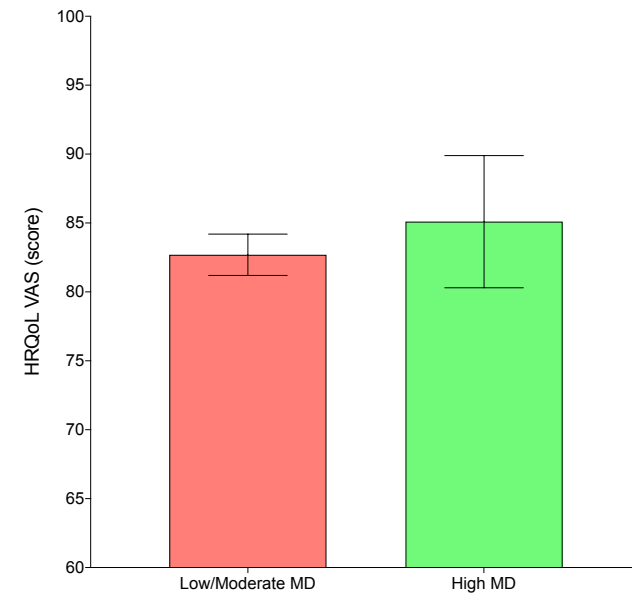

Supplement: Supplementary file 1 [file nutrients-15-00677-s001.zip › Figure S2.pdf]

**A**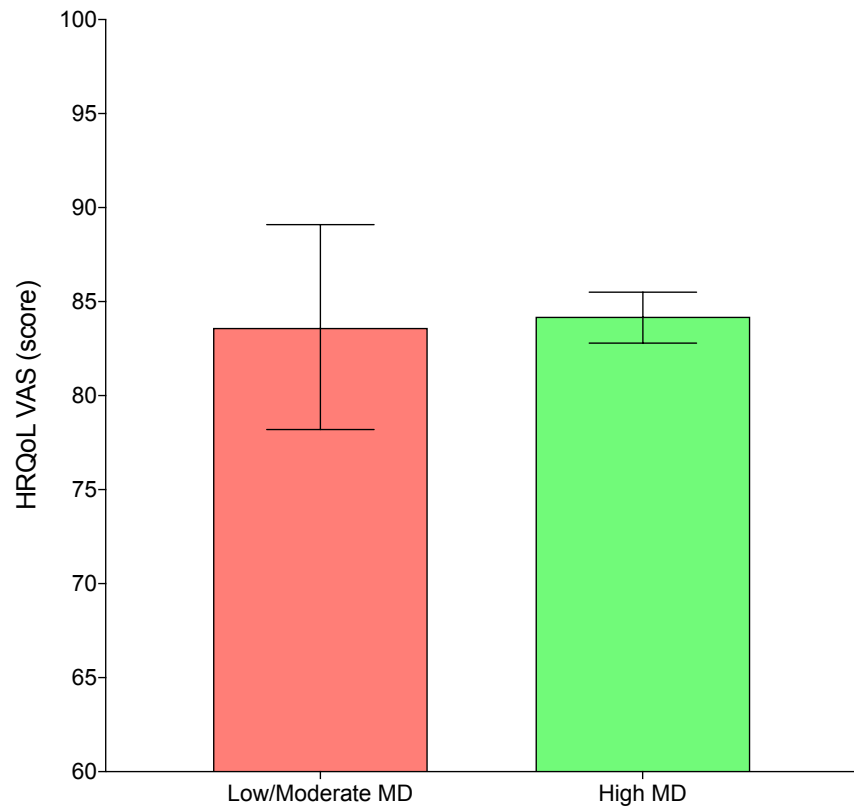**B**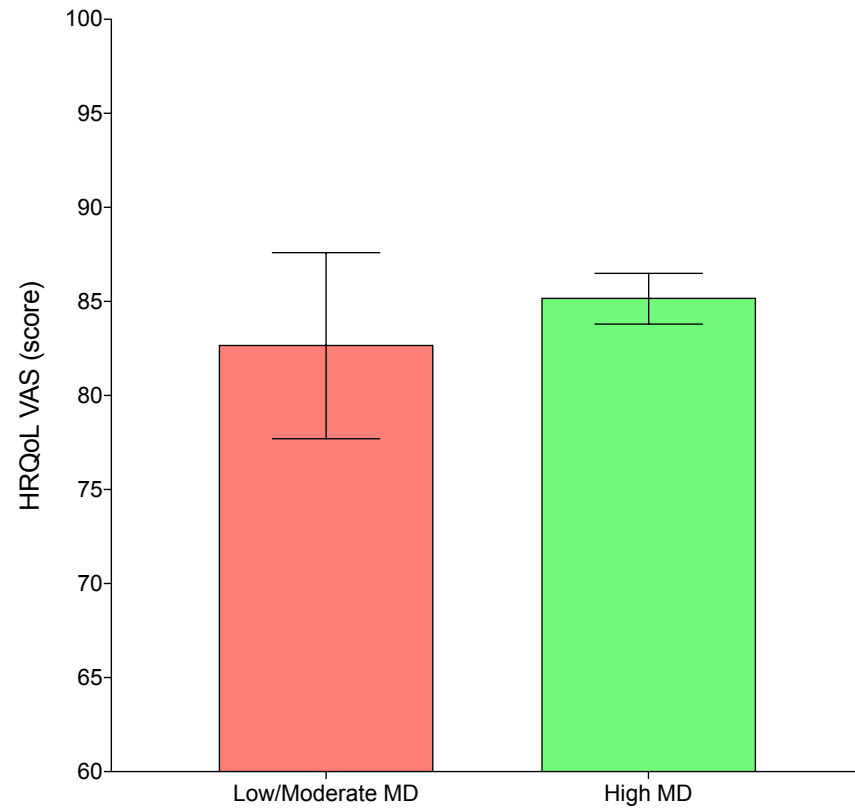

Supplement: Supplementary file 1 [file nutrients-15-00677-s001.zip › Figurea S1.pdf]
